# On azimuthally propagating equatorial atmospheric waves

**DOI:** 10.1007/s00605-022-01741-x

**Published:** 2022-07-08

**Authors:** Calin I. Martin

**Affiliations:** Faculty of Mathematics, University of Vienna, Oskar-Morgenstern-Platz 1, 1090 Vienna, Austria

**Keywords:** Atmospheric flow, Standing wave, Rotating spherical coordinates, 86A10, 35Q86

## Abstract

We investigate the existence of solutions to a recent model for large-scale equatorial waves, derived recently by an asymptotic method driven by the thin-shell approximation of the Earth’s atmosphere in rotating spherical coordinates.

## Introduction

Due to the Earth’s rotation, geophysical waves in equatorial regions propagate typically in the azimuthal direction, since the change of sign of the Coriolis force across the Equator produces an effective waveguide, forcing a practically azimuthal flow propagation (see the discussions in [1, 2]). While in the geophysical research literature there is a preference for flat-space geometry by means of the *f*-plane approximation (see [9, 14]), and this approach still provides a lot of insight into the equatorial ocean dynamics (see [2, 12] and references therein) due to the large-scale nature of atmospheric flows, in studying them it is better to take into account curvature effect by working in rotating spherical coordinates (see the discussions in [4, 6, 15, 17]). While recent studies of oceanic flows in rotating spherical coordinates are available [3, 7, 8, 11, 13], note that the methods used in these papers do not apply to atmospheric flows. The main differences are due to the fact that the temperature forcing is a key factor for atmospheric flows (and plays a more modest role in the ocean) and in the ease at which density and pressure vary in the compressible atmosphere – see the discussions in [4, 6]. Another fundamental difference in the two geophysical flow structures – oceanic and atmospheric – comes about because for the atmosphere, the flow necessarily involves a perturbation away from a background state, whereas the ocean does not (see the discussion in [10]). Fortunately, however, the perturbation is not based on some suitable amplitude parameter (as might be expected), rather it is simply the thin-shell parameter, and so this is valid for atmospheric motions of any finite size, bringing together all the leading-order dynamics and thermodynamics, at the same order and without any additional approximations (see [5]).

In this paper we investigate a model for the propagation of atmospheric waves derived recently [3], considering the issue of the existence of solutions in equatorial regions, where, as pointed out above, we can take advantage of the fact that the direction of propagation is azimuthal to simplify the dynamics. We use a Fourier mode decomposition to gain insight into the dynamics.

## Preliminaries

Adhering to the point of view of [4] we regard the atmosphere to be a compressible, viscous fluid. Therefore, we use the Navier-Stokes and mass conservation equations of fluid dynamics, allowing for variable density, coupled to an equation of state and a suitable version of the first law of thermodynamics. When formulating the governing equations we take into account that the shape of the Earth is (essentially) that of an oblate spheroid. In atmospheric science, it is customary to approximate the oblate spheroid by an ellipsoid obtained by rotating an ellipse, whose center coincides with the center of the Earth, about its semi-minor polar axis (of length $d'_P\approx 6357\hbox { km}$), with a semi-major equatorial axis of length $d'_E\approx 6378\hbox { km}$. (We use the prime notation to refer to physical, dimensional, variables. After suitable non-dimensionalization, the prime notation will be removed.)

**Fig. 1:**
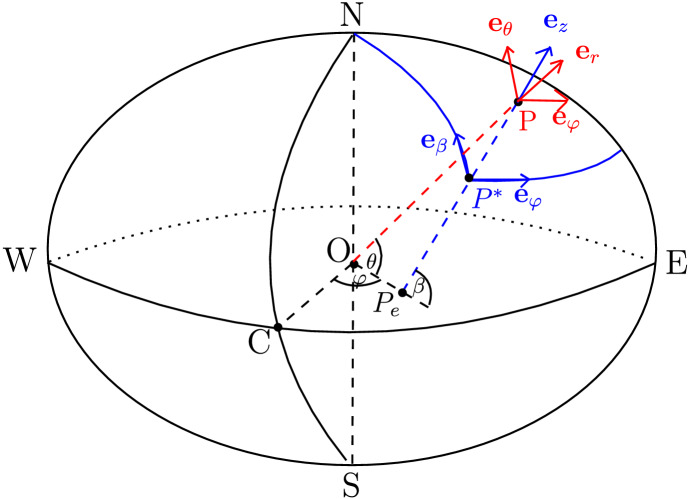
Representation of a point *P* in the atmosphere (away from the polar axis) using the hybrid spherical-geopotential rotating coordinate system $(\varphi ,\theta ,z')$ which is derived from the spherical system $({\mathbf {e}}_{\varphi },{\mathbf {e}}_{\theta }, {\mathbf {e}}_r)$ and the geopotential system $({\mathbf {e}}_{\varphi },{\mathbf {e}}_{\beta }, {\mathbf {e}}_z)$. We have denoted with $\varphi$ and $\theta$ the longitude and the geocentric latitude of *P*, respectively, and with $\beta$ the geodetic latitude of the projection $P^{*}$ of *P* on the ellipsoidal geoid. The unit vector ${\mathbf {e}}_z$ points vertically upwards along the normal $PP^{*}$ to the geoid (which intersects the equatorial plane in the point $P_e$), while $({\mathbf {e}}_{\theta },{\mathbf {e}}_r)$ are obtained by rotating the unit vectors $({\mathbf {e}}_{\beta },{\mathbf {e}}_z)$ by the angle $\beta -\theta$, in the plane of fixed longitude $\varphi$

The longitude $\varphi$ and the geodetic latitude $\beta$ are used to define Cartesian coordinates

$$\begin{aligned} (X',Y',Z')=\frac{d'_E}{\sqrt{1-{\mathfrak {e}}^2\sin ^2\beta }}\left( \cos \beta \cos \varphi ,\cos \beta \sin \varphi , (1-{\mathfrak {e}}^2)\sin \beta \right) \end{aligned}$$

where

$$\begin{aligned} {\mathfrak {e}}=\sqrt{1-\left( \frac{d'_P}{d'_E}\right) ^2}\approx 0.081 \end{aligned}$$

denotes the eccentricity. We associate to the ellipsoid (rotating with constant angular speed $\Omega '\approx 7.29\times 10^{-5}$ rad $\hbox {s}^{-1}$) the coordinate system $(\varphi ,\beta , z')$, where $z'$ is the vertical distance up from the surface of the ellipsoid. The unit tangent vectors at the surface of the ellipsoid are $({\mathbf {e}}_{\varphi },{\mathbf {e}}_{\beta },{\mathbf {e}}_z)$: ${\mathbf {e}}_{\varphi }$ points from West to East along the geodetic parallel, ${\mathbf {e}}_{\beta }$ from South to North along the geodetic meridian and ${\mathbf {e}}_z$ points upwards, cf. Fig. 1.

This system is valid everywhere, except along the direction of the polar axis. More details about the geometry of this system are given in [4] where it is advocated for the passage from spherical coordinates $(\varphi , \theta ,r')$ to the hybrid spherical-geopotential coordinates $(\varphi , \theta , z')$ coupled with a transformation of the velocity vector and the gravity term. The advantage of the spherical-geopotential hybrid rotating coordinate system over the spherical potential approximation ([16]) is that, in the former, the formulation retains the details of the curved-space geometry of the Earth, and the leading-order (geometrical) correction terms apply to the background state of the atmosphere but do not interact (in the leading-order perturbation) with the dynamics of the atmosphere.

To render the equations of motion in a form that is relevant for a discussion of atmospheric flows we will perform a non-dimensionalization. For this purpose, we will use an appropriate scale length (taken to be the maximum height of the troposphere $H'\approx 16\hbox { km}$ at the Equator) and an associated speed scale $\Omega ' H' (\approx 1.2\hbox { m }\hbox {s}^{-1}$). Here and in what follows we use the “ $'$ ” notation for physical quantities. The non-dimensional quantities appear without the prime notation. For instance $(u',v',w')$ denotes the velocity field in spherical coordinates while (*u*, *v*, *w*) is a non-dimensional velocity with *w* normal and (*u*, *v*) tangential to the ellipsoidal geoid. The pressure, $p^{\prime }$, and the temperature, $T^{\prime }$, will also be non-dimensionalized, the resulting non-dimensional quantities being denoted with *p* and *T*, respectively. Moreover, ${\bar{\rho }}'\approx 0.8\hbox { kg }\hbox {m}^{-3}$ denotes the density and ${\bar{\mu }}'\approx 2\times 10^{-2}\hbox { m}^{-1}\hbox { s}^{-1}$ denotes the dynamic eddy viscosity, which is assumed to vary only in the radial direction as in [4]. More precisely, we define

$$\begin{aligned} \begin{aligned} z'&=\varepsilon z\,\,{\mathrm{where}}\,\, \varepsilon =\frac{H'}{d'_E}\\ t'&=\frac{1}{\Omega '}t\\ (u',v',w')&=\Omega ' H'(u,v\cos (\beta -\theta )+kw\sin (\beta -\theta ),kw\cos (\beta -\theta )\\&\qquad -v\sin (\beta -\theta ))\\ \rho '&={\bar{\rho }}'\rho ,\,\,\mu '={\bar{\mu }}'\mu \\ p'&={\bar{\rho }}'(\Omega ' d'_E)^2p,\,\,T'=\frac{(\Omega 'd'_E)^2}{{\mathfrak {R}}'}T \end{aligned} \end{aligned}$$

where ${\mathfrak {R}}'\approx 287$ $\hbox {m}^2$ $\hbox {s}^{-2}$ $K^{-1}$ is the universal gas constant and the constant *k* measures the size of the velocity component normal to the ellipsoid.

The transformation from $r'$ to $z'$ shows (cf. [4]) that the viscous terms appearing in the Navier-Stokes equation satisfy

2.1 $$\begin{aligned} \mu =m(z)+O({\mathfrak {e}}^6,\varepsilon {\mathfrak {e}}^4,\varepsilon ^2{\mathfrak {e}}^2,\varepsilon ^3). \end{aligned}$$

With the previous considerations in mind we invoke in the following (cf. [6]) a suitable approximation of the Navier-Stokes equations by considering small $\varepsilon$ and small $\delta ={\mathfrak {e}}^2$, where the latter parameter is indicative of the effects of small deviations of the ellipsoid from the sphere. More precisely, denoting

$$\begin{aligned} \begin{aligned}&\frac{D}{Dt}=\frac{\partial }{\partial t}+\varepsilon \frac{u}{\cos \theta }\frac{\partial }{\partial \varphi }+\varepsilon v \frac{\partial }{\partial \theta }+\varepsilon w \frac{\partial }{\partial z}\\&\Delta (\theta )=\frac{1}{2}\sin \theta \cos \theta ,\,\,D(\theta )=\left( 1-\frac{3}{2}\sin ^2\theta \right) \sin \theta \cos \theta \end{aligned} \end{aligned}$$

the governing equations we will work with are

2.2a $$\begin{aligned}&\varepsilon \rho \frac{\partial u}{\partial t}-2\varepsilon \rho v\sin \theta =-\frac{1-\varepsilon z+\delta \Delta }{\cos \theta }\frac{\partial p}{\partial \varphi }+\frac{\varepsilon }{R_e}\frac{\partial }{\partial z}\left( m\frac{\partial u}{\partial z}\right) +O(\varepsilon ^2,\varepsilon \delta ,\delta ^2) \end{aligned}$$

2.2b $$\begin{aligned}&\varepsilon ^2\rho \frac{\partial v}{\partial t}+2\varepsilon ^2\rho u\sin \theta +\varepsilon \rho (1+\varepsilon z-\delta \Delta )\sin \theta \cos \theta \end{aligned}$$

2.2c $$\begin{aligned}&\quad =-(1-\varepsilon z+\delta \Delta )\left( \varepsilon \frac{\partial p}{\partial \theta } +(2\delta \Delta +\delta ^2 D)\frac{\partial p}{\partial z}\right) \nonumber \\&\qquad \quad -2\delta \Delta \rho g[1+\delta (\sin \theta +\cos \theta )\cos \theta -3\varepsilon z]+\frac{\varepsilon ^2}{R_e}\frac{\partial }{\partial z}\left( m\frac{\partial v}{\partial z}\right) \nonumber \\&\qquad \quad +O(\varepsilon ^3,\varepsilon ^2\delta ,\varepsilon \delta ^2) -\varepsilon \rho \cos ^2\theta \nonumber \\&\quad =-\frac{\partial p}{\partial z}-\rho g(1-2\varepsilon z+2\delta \Delta )+O(\varepsilon ^2, \varepsilon \delta ,\delta ^2), \end{aligned}$$

2.2d $$\begin{aligned}&\frac{D\rho }{Dt}+\varepsilon \rho \left[ \frac{1}{\cos \theta }\left( \frac{\partial u}{\partial \varphi }+\frac{\partial }{\partial \theta }(v\cos \theta )\right) +\frac{\partial w}{\partial z}\right] =O(\varepsilon ^2, \varepsilon \delta ,\delta ^2), \end{aligned}$$

2.2e $$\begin{aligned}&p=\rho T \end{aligned}$$

and

2.2f $$\begin{aligned} c_p\frac{DT}{Dt}-\varepsilon \kappa \frac{\partial ^2 T}{\partial z^2}-\frac{1}{\rho }\frac{Dp}{Dt}=\varepsilon Q(\varphi ,\theta ,z;\varepsilon ,\delta )+O(\varepsilon ^2,\varepsilon \delta ,\delta ^2), \end{aligned}$$

where the last equation is the first law of thermodynamics with *Q* being the non-dimensional hear sources (or sinks), expressing the change of total energy due to any heat exchanges. We have introduced above the non-dimensional constants (held fixed throughout the non-dimensionalization)

2.3 $$\begin{aligned} \begin{aligned}&c_p=\frac{c'_p}{{\mathfrak {R}}'}\approx 5.25,\,\,\kappa =\frac{\nu ' c'_p}{{\mathfrak {R}}'\Omega ' H'^2}=6\times 10^{-9},\\&R_e=\frac{{\bar{\rho }}'\Omega ' H'^2}{{\bar{\mu }}'}\approx 7\times 10^5,\,\,g=\frac{g' H'}{(\Omega ' d'_E)^2}\approx 0.72. \end{aligned} \end{aligned}$$

To search for solutions of the (rather) complicated system (2.2a)–(2.2f) we perform an asymptotic expansion

$$\begin{aligned} {\mathfrak {U}}(\varphi ,\theta ,z,t; \varepsilon ,\delta )\sim {\mathfrak {U}}_0(\varphi ,\theta ,z)+\varepsilon {\mathfrak {U}}_1(\varphi ,\theta ,z,t)+\delta \tilde{{\mathfrak {U}}}_1(\varphi ,\theta ,z,t), \end{aligned}$$

where ${\mathfrak {U}},{\mathfrak {U}}_n$ and $\tilde{{\mathfrak {U}}}_n$ stand for each of the variables $u,v,w,p,\rho , T$. Under the assumption that the boundary and initial conditions and the heat source term are consistent with the previous asymptotic expansion we obtain that at the leading-order, $O(\varepsilon ^0)$, the problem is time-independent. More precisely, setting

$$\begin{aligned} L_0:=\left( \frac{u_0}{\cos \theta }\frac{\partial }{\partial \varphi }+v_0\frac{\partial }{\partial \theta }+w_0\frac{\partial }{\partial z}\right) \end{aligned}$$

we obtain that the leading order approximation is given as the problem

2.4 $$\begin{aligned} \begin{aligned} \frac{\partial p_0}{\partial \varphi }&=0,\\ \frac{\partial p_0}{\partial \theta }&=-\rho _0\sin \theta \cos \theta ,\\ \frac{\partial p_0}{\partial z}&=-\rho _0 g,\\ \frac{\partial \rho _0}{\partial t}&=0,\\ p_0&=\rho _0 T_0,\\ Q_0(\varphi ,\theta ,z)&=c_pL_0(T_0)-\kappa \frac{\partial ^2 T_0}{\partial z^2}-\frac{1}{\rho _0}L_0(p_0), \end{aligned} \end{aligned}$$

with solution

2.5 $$\begin{aligned} \begin{aligned} T_0&=A-\frac{1}{c_p}\left( gz-\frac{1}{2}\cos ^2\theta \right) ,\\ p_0&=B\left[ A-\frac{1}{c_p}\left( gz-\frac{1}{2}\cos ^2\theta \right) \right] ^{c_p},\\ \rho _0&=B\left[ A-\frac{1}{c_p}\left( gz-\frac{1}{2}\cos ^2\theta \right) \right] ^{c_p-1}. \end{aligned} \end{aligned}$$

The important time-dependence appears at order $O(\varepsilon )$ for which the equations assume the form

2.6a $$\begin{aligned}&\rho _0\frac{\partial u_0}{\partial t}-2\rho _0 v_0\sin \theta =-\frac{1}{\cos \theta }\left( \frac{\partial p_1}{\partial \varphi }-z\frac{\partial p_0}{\partial \varphi }\right) +\frac{1}{R_e}\frac{\partial }{\partial z}\left( m\frac{\partial u_0}{\partial z}\right) , \end{aligned}$$

2.6b $$\begin{aligned}&\rho _0\frac{\partial v_0}{\partial t}+2\rho _0 u_0\sin \theta +(\rho _1+z\rho _0)\sin \theta \cos \theta = -\left( \frac{\partial p_1}{\partial \theta }-z\frac{\partial p_0}{\partial \theta }\right) +\frac{1}{R_e}\frac{\partial }{\partial z}\left( m\frac{\partial v_0}{\partial z}\right) , \end{aligned}$$

2.6c $$\begin{aligned}&-\rho _0\cos ^2\theta =-\frac{\partial p_1}{\partial z}-g(\rho _1-2z\rho _0), \end{aligned}$$

2.6d $$\begin{aligned}&\cos \theta \frac{\partial \rho _1}{\partial t}+\frac{\partial }{\partial \varphi }(\rho _0 u_0)+\frac{\partial }{\partial \theta } (\rho _0 v_0\cos \theta )+\frac{\partial }{\partial z}(\rho _0 w_0\cos \theta )=0, \end{aligned}$$

2.6e $$\begin{aligned}&p_1=\rho _0 T_1+\rho _1 T_0, \end{aligned}$$

2.6f $$\begin{aligned}&c_p\frac{\partial T_1}{\partial t}-\frac{1}{\rho _0}\frac{\partial p_1}{\partial t}={\mathcal {Q}}_0(\varphi ,\theta ,z,t), \end{aligned}$$

where

$$\begin{aligned} Q\sim Q_0(\varphi ,\theta ,z)+{\mathcal {Q}}_0(\varphi ,\theta ,z,t)+\delta {\mathcal {Q}}_1(\varphi ,\theta ,z,t) \end{aligned}$$

with $Q_0\equiv 0$ for the troposphere.

A further transformation of the above system is necessary so that the finding of exact solutions becomes possible. To this end we set

$$\begin{aligned} {\mathcal {q}}_0(\varphi , \theta , z,t)=\int _0^t{\mathcal {Q}}_0(\varphi ,\theta ,z,s)\,ds \end{aligned}$$

which implies ${\mathcal {Q}}_0=\frac{\partial {\mathcal {q}}_0}{\partial t}$. Hence, integrating now Eq. (2.6f) we obtain

$$\begin{aligned} c_p T_1-\frac{p_1}{\rho _0}={\mathcal {q}}_0(\varphi ,\theta ,z,t)+A_1(\varphi ,\theta ,z), \end{aligned}$$

where $A_1(\varphi ,\theta ,z)$ is an arbitrary function which is determined by the initial data on the perturbation temperature and pressure. Moreover, setting

$$\begin{aligned} P_1=\int _0^z\left( \frac{{\mathcal {q}}_0(\varphi ,\theta ,\xi ,t)+A_1(\varphi ,\theta ,\xi )}{T_0(\theta ,\xi )}\right) \, d\xi , \end{aligned}$$

we obtain from (2.6c) the relation

2.7 $$\begin{aligned} p_1=\rho _0[(gz^2+z\cos ^2\theta )+g P_1 +B_1], \end{aligned}$$

where $B_1(\varphi ,\theta ,t)$ is an arbitrary function determined by the perturbation pressure on the ground. The thermodynamic properties of the atmosphere are completed by the relations

2.8 $$\begin{aligned} T_1=\frac{1}{c_p}\left( \frac{p_1}{\rho _0}+{\mathcal {q}}_0+A_1\right) ,\quad \rho _1=\frac{1}{c_p T_0}\Big [(c_p-1)p_1-({\mathcal {q}}_0+A_1)p_0\Big ] \end{aligned}$$

obtained from (2.6e) and (2.6f). Putting now

2.9 $$\begin{aligned} F_1(\varphi , \theta , z,t)=gP_1(\varphi , \theta , z,t)+B_1(\varphi ,\theta , t) \end{aligned}$$

the system (2.6) can be written as

2.10a $$\begin{aligned}&\rho _0\frac{\partial u_0}{\partial t}-2\rho _0 v_0\sin \theta =-\frac{\rho _0}{\cos \theta }\frac{\partial F_1}{\partial \varphi } +\frac{1}{R_e}\frac{\partial }{\partial z}\left( m\frac{\partial u_0}{\partial z}\right) , \end{aligned}$$

2.10b $$\begin{aligned}&\rho _0\frac{\partial v_0}{\partial t}+2\rho _0 u_0\sin \theta =-\rho _0\frac{\partial F_1}{\partial \theta }+ \frac{\rho _0\sin \theta \cos \theta }{g}\frac{\partial F_1}{\partial z}+\frac{1}{R_e}\frac{\partial }{\partial z}\left( m\frac{\partial v_0}{\partial z}\right) , \end{aligned}$$

2.10c $$\begin{aligned}&\frac{\partial }{\partial \varphi }(\rho _0 u_0)+\frac{\partial }{\partial \theta } (\rho _0 v_0\cos \theta )+\frac{\partial }{\partial z}(\rho _0 w_0\cos \theta )=\frac{\rho _0\cos \theta }{g}\left( \frac{\partial ^2 F_1}{\partial z\partial t}-\frac{c_p-1}{c_p}\frac{g}{T_0}\frac{\partial F_1}{\partial t}\right) . \end{aligned}$$

Motivated by the shape of solutions (2.5) we perform the change of variables $\zeta =gz-\frac{1}{2}\cos ^2\theta$ which transforms from the variables $(\varphi ,\theta ,z,t)$ to $(\varphi ,\theta ,\zeta ,t)$ and so the previous system becomes

2.11a $$\begin{aligned}&\rho _0\frac{\partial u_0}{\partial t}-2\rho _0 v_0\sin \theta =-\frac{\rho _0}{\cos \theta }\frac{\partial F_1}{\partial \varphi }+\frac{g^2}{R_e}\frac{\partial }{\partial \zeta }\left( M\frac{\partial u_0}{\partial \zeta }\right) , \end{aligned}$$

2.11b $$\begin{aligned}&\rho _0\frac{\partial v_0}{\partial t}+2\rho _0 u_0\sin \theta =-\rho _0\frac{\partial F_1}{\partial \theta }+\frac{g^2}{R_e}\frac{\partial }{\partial \zeta }\left( M\frac{\partial v_0}{\partial \zeta }\right) , \end{aligned}$$

2.11c $$\begin{aligned}&\frac{\partial }{\partial \varphi }(\rho _0 u_0)+\frac{\partial }{\partial \theta } (\rho _0 v_0\cos \theta )+\frac{\partial }{\partial \zeta }(g\rho _0 w_0\cos \theta +\rho _0 v_0\sin \theta \cos ^2\theta )=\cos \theta \, \frac{\partial ^2(\rho _0 F_1)}{\partial \zeta \partial t}, \end{aligned}$$

where $$m(z):=M(\zeta +\frac{1}{2}\cos ^2\theta )$$.

Owing now to the large Reynolds numbers (cf. the discussion in [5]) we pass to study the inviscid limit of the system in (2.11). In doing so we denote

$$\begin{aligned} \rho _0(u_0,v_0,w_0,F_1)=(U,V,W,F), \end{aligned}$$

and so obtain the model of the propagation of waves in the troposphere

2.12a $$\begin{aligned}&\frac{\partial U}{\partial t} - 2V \sin \theta = - \, \frac{1}{\cos \theta }\,\frac{\partial F}{\partial \varphi }\,, \end{aligned}$$

2.12b $$\begin{aligned}&\frac{\partial V}{\partial t} + 2 U\sin \theta = - \,\frac{\partial F}{\partial \theta } \,, \end{aligned}$$

2.12c $$\begin{aligned}&\frac{\partial U}{\partial \varphi } + \frac{\partial }{\partial \theta }\,(V\cos \theta ) + \frac{\partial }{\partial \zeta }\,(V\sin \theta \cos ^2\theta + gW\cos \theta ) = \cos \theta \,\frac{\partial ^2 F}{\partial \zeta \partial t}\,. \end{aligned}$$

The latter model was derived recently [5] in the inviscid limit of the governing equations for atmospheric flows in non-polar regions. In (2.12a)–(2.12c) the vector (*U*, *V*, *W*) is the non-dimensional velocity (with *U* zonal velocity, *V* meridional velocity and *W* vertical velocity, *t* being time, $\theta \in (-\frac{\pi }{2},\frac{\pi }{2})$ the angle of latitude and $\varphi \in [0,2\pi )$ the angle of longitude) scaled by the background density, so that the explicit dependence on the background state of the the atmosphere is already accounted for (see the discussion in [5]). The system (2.12a)–(2.12c) provides a complete description of the (inviscid) velocity field at leading order, for a given forcing *F*, which represents the perturbation to the thermodynamic state, encompassing the identification of heat sources. Concerning the neglection of viscous effects, note that this is reasonable above the atmospheric boundary layer (see the discussion in [9]), so that (2.12a)–(2.12c) captures the leading-order dynamics of time-dependent atmospheric flows in the upper troposphere.

## A Fourier modes existence approach for equatorial waves

As pointed out in the introduction, in equatorial regions the flows is typically azimuthal, so that we investigate the system (2.12a)–(2.12c) under the assumption of a vanishing meridional velocity component ($V \equiv 0$):

3.1 $$\begin{aligned}&\frac{\partial U}{\partial t}=-\frac{1}{\cos \theta }\frac{\partial F}{\partial \varphi } \end{aligned}$$

3.2 $$\begin{aligned}&2U\sin \theta =-\frac{\partial F}{\partial \theta } \end{aligned}$$

3.3 $$\begin{aligned}&\frac{\partial U}{\partial \varphi }+g\cos \theta \frac{\partial W}{\partial \zeta }=\cos \theta \frac{\partial ^2 F}{\partial \zeta \partial t} \end{aligned}$$

Setting $f=U\cos \theta$ we proceed to eliminate the forcing *F* between the first two equations anove. We obtain

3.4 $$\begin{aligned} f_{t\theta }=2 f_{\varphi }\tan \theta \end{aligned}$$

With the Fourier series ansatz

$$\begin{aligned} f=\sum _{n\in {\mathbb {Z}}} f_n(\theta , \zeta ,t) e^{in\varphi } \end{aligned}$$

we have that each $f_n\,(n\ne 0)$ satisfies

$$\begin{aligned} (f_n)_{t\theta }=2i n (\tan \theta ) f_n(\theta ,\zeta ,t), \end{aligned}$$

while $$(f_0)_{t\theta }\equiv 0$$. Setting

3.5 $$\begin{aligned} f_n(\theta ,\zeta ,t)=e^{i\omega _n t}g_n(\theta ,\zeta ) \end{aligned}$$

we conclude that $g_n$ satisfies the equation

3.6 $$\begin{aligned} \omega _n (g_n)_{\theta }(\theta ,\zeta )=2n(\tan \theta ) g_n(\theta ,\zeta ), \end{aligned}$$

which, upon integration, delivers

3.7 $$\begin{aligned} g_n(\theta ,\zeta )=c_n(\zeta )\left( \frac{1}{\cos \theta }\right) ^{\frac{2n}{\omega _n}}, \end{aligned}$$

where $$c_n(\zeta )=g_n(0,\zeta )$$. Hence

3.8 $$\begin{aligned} U(\theta , \zeta , \varphi ,t)=\frac{1}{\cos \theta }\left( f_0(\zeta )+ {\sum }_{n\in {\mathbb {Z}}^{*}}c_n(\zeta ) \left( \frac{1}{\cos \theta }\right) ^{\frac{2n}{\omega _n}}e ^{i(\omega _n t+n\varphi )}\right) . \end{aligned}$$

To determine $\omega _n$ let

3.9 $$\begin{aligned} F=\sum _{n\in {\mathbb {Z}}} F_n(\theta ,\zeta ,t) e^{in\varphi } \end{aligned}$$

be the Fourier series representation of *F*. Then from Eq. (3.1) we obtain that for each $n\ne 0$ it holds that

3.10 $$\begin{aligned} F_n=-c_n(\zeta )\frac{\omega _n}{n}\left( \frac{1}{\cos \theta }\right) ^{\frac{2n}{\omega _n}} e^{i\omega _n t}, \end{aligned}$$

and so

3.11 $$\begin{aligned} (F_n)_{\theta }=-c_n(\zeta )\frac{\omega _n}{n}\left( \frac{1}{\cos \theta }\right) ^{\frac{2n}{\omega _n}} (\tan \theta ) e^{i\omega _n t}. \end{aligned}$$

On the other hand, utilizing (3.2) and (3.8) we have

3.12 $$\begin{aligned} (F_n)_{\theta }=-2c_n(\zeta )\left( \frac{1}{\cos \theta }\right) ^{\frac{2n}{\omega _n}} (\tan \theta ) e^{i\omega _n t}. \end{aligned}$$

Comparing now (3.11) and (3.12) we infer that $\omega _n=2n$ for all $n\in {\mathbb {Z}}^{*}$ and $\omega _0=0$. Consequently,

3.13 $$\begin{aligned} U(\theta , \zeta ,\varphi ,t)=\frac{f_0(\zeta )}{\cos \theta }+ \frac{1}{\cos ^2\theta }\sum _{n\in {\mathbb {Z}}^{*}}c_n(\zeta )e^{2ni t}\cdot e^{in\varphi }, \end{aligned}$$

and

3.14 $$\begin{aligned} F_n(\theta ,\zeta ,t)=\left\{ \begin{aligned}&-\frac{2c_n(\zeta )}{\cos \theta } e^{i 2n t}=-\frac{2f_n(0,\zeta ,t)}{\cos \theta }\quad {{\mathrm{if}}}\quad n\ne 0,\\&f_0(\zeta )\ln (\cos ^2\theta ) \quad {{\mathrm{if}}}\quad n=0, \end{aligned}\right. \end{aligned}$$

by the ansatz (3.5) for $f_n$. Moreover, writing

$$\begin{aligned} W(\theta ,\zeta ,\varphi ,t)=\sum _{n\in {\mathbb {Z}}}W_n (\theta ,\zeta ,t)e^{in\varphi } \end{aligned}$$

we find from (3.3) that

3.15 $$\begin{aligned} (W_n)_{\zeta }=\frac{-in}{g\cos \theta }\left( 4c_n^{'}(\zeta )+\frac{c_n(\zeta )}{\cos ^2\theta }\right) e^{i2nt}\quad {\mathrm{if}}\quad n\ne 0, \end{aligned}$$

which, since $W_n\Big |_{\zeta =0}=0$, implies that

3.16 $$\begin{aligned} W_n(\theta ,\zeta , t)=\frac{-in}{g\cos \theta }\left( 4c_n(\zeta )-4c_n(0)+\frac{1}{\cos ^2\theta }\int _0^{\zeta }c_n({\tilde{\zeta }})\,d{\tilde{\zeta }}\right) e^{i2nt}\quad {\mathrm{if}}\quad n\ne 0 \end{aligned}$$

where, we recall, $c_n(\zeta )=g_n(\theta =0, \zeta )$. Also from (3.3) we have that $(W_0)_{\zeta }\equiv 0$ and, since $W_0\big |_{\zeta =0}$ we conclude that $W_0\equiv 0$.

### Remark 3.1

Formula (3.16) indicates that the vertical velocity increases with increasing latitude $\theta$, observation which is in agreement with the results of [6].

## Data Availability

The material presented here has no associated data.
